# Small Drugs, Huge Impact: The Extraordinary Impact of Antisense Oligonucleotides in Research and Drug Development

**DOI:** 10.3390/molecules27020536

**Published:** 2022-01-15

**Authors:** Anais M. Quemener, Maria Laura Centomo, Scott L. Sax, Riccardo Panella

**Affiliations:** 1 University Rennes, CNRS, IGDR (Institute of Genetics and Development of Rennes)-UMR 6290, F-35000 Rennes, France; anais.quemener@univ-rennes1.fr; 2 Department of Oncology, University of Turin, 10124 Turin, Italy; marialaura.centomo@dri.edu; 3 Center for Genomic Medicine, Desert Research Institute, Reno, NV 89512, USA; scott.sax@dri.edu

**Keywords:** ASO, RNA medicine, non-coding RNA, COVID-19, SARS-CoV-2, drug development, RNA therapy, RNA, drug design, drug, therapy, miRNA, drug discovery, precision medicine

## Abstract

Antisense oligonucleotides (ASOs) are an increasingly represented class of drugs. These small sequences of nucleotides are designed to precisely target other oligonucleotides, usually RNA species, and are modified to protect them from degradation by nucleases. Their specificity is due to their sequence, so it is possible to target any RNA sequence that is already known. These molecules are very versatile and adaptable given that their sequence and chemistry can be custom manufactured. Based on the chemistry being used, their activity may significantly change and their effects on cell function and phenotypes can differ dramatically. While some will cause the target RNA to decay, others will only bind to the target and act as a steric blocker. Their incredible versatility is the key to manipulating several aspects of nucleic acid function as well as their process, and alter the transcriptome profile of a specific cell type or tissue. For example, they can be used to modify splicing or mask specific sites on a target. The entire design rather than just the sequence is essential to ensuring the specificity of the ASO to its target. Thus, it is vitally important to ensure that the complete process of drug design and testing is taken into account. ASOs’ adaptability is a considerable advantage, and over the past decades has allowed multiple new drugs to be approved. This, in turn, has had a significant and positive impact on patient lives. Given current challenges presented by the COVID-19 pandemic, it is necessary to find new therapeutic strategies that would complement the vaccination efforts being used across the globe. ASOs may be a very powerful tool that can be used to target the virus RNA and provide a therapeutic paradigm. The proof of the efficacy of ASOs as an anti-viral agent is long-standing, yet no molecule currently has FDA approval. The emergence and widespread use of RNA vaccines during this health crisis might provide an ideal opportunity to develop the first anti-viral ASOs on the market. In this review, we describe the story of ASOs, the different characteristics of their chemistry, and how their characteristics translate into research and as a clinical tool.

## 1. Introduction

Antisense oligonucleotide (ASO) drug development is emerging as a powerful tool for targeting RNAs, and has revolutionized the field of drug discovery. A rational drug design for any disease requires the identification of a suitable target and the development of a drug with a specific affinity for that target.

The main goal is to understand the molecular mechanisms responsible for diseases and to investigate whether downregulating or activating a certain component of the molecular pathway of a disease would produce the desired biological effects. This would then be useful to the development of a new drug to treat that disease.

The ability of an ASO to specifically recognize any RNA (via Watson–Crick base-pairing) means that the only information required for their synthesis is the target RNA sequence. This is now possible given the publication of the human genome sequence. Therefore, it seems straightforward to design a low-cost, highly specific lead compound to selectively target almost any gene (both coding and non-coding). As such, ASOs represent a very powerful target-directed approach for identification and validation in vitro, and subsequentially in vivo. This can be accomplished in a rapid manner, limiting the use of KO gene mouse models. It also provides an opportunity to test a therapeutic approach in the early stage of a project. Moreover, ASOs are highly valuable as novel and personalized therapeutic strategies to treat a wide range of diseases linked to dysregulated gene expression (including rare diseases where conventional therapies are not available [1]). More importantly, ASOs can be used as structural blockers, aiming to mask a specific sequence and make it inaccessible for other cellular components like transcriptional factors or spliceosome members. In this way, complex biological processes might be modulated in ways that have limited previous approaches involving “traditional” drugs.

ASOs are able to affect gene expression in several ways. Specifically, they can cause RNA cleavage or RNA steric blockage in both the cytoplasm and the nucleus [2], depending on their chemical make-up and the location of hybridization. For specific knockdowns which silence gene expression, ASOs with RNase H activity are usually employed. RNase H is able to recognize any DNA/RNA heteroduplex longer that 5 nucleotides and once this structural recognition is in place, RNase H is able to digest the RNA part of the heteroduplex. The induction of RNase H endonuclease leads to the cleavage and degradation of the mRNA target while leaving the ASO intact and free to bind to another copy of RNA [3]. This supplies a long-lasting effect, making it possible to use them in a micro or nano molar concentration and to reduce the number of treatments over time, thus reducing possible side effects without affecting the efficacy of the treatment. There are ASOs based on steric hindrance which is related to their binding affinity. In fact, steric block ASO are designed against specific sequences within a target transcript to mask them, thereby interfering with different mechanisms. These include (1) translation inhibition by binding AUG start codon which prevent ribosome assembly, (2) pre-mRNA destabilization preventing its capping or polyadenylation at 3’ or 5’ respectively, (3) activation of protein expression interfering with upstream open reading frames (uORFs) that negatively regulate translation, and (4) modulation of alternative splicing to either promote exon skipping or exon exclusion and move the balance between different isoforms in favor of a specific genetic product [4,5]. Typically, such splice corrections are the most widely used application of steric block ASOs in order to remove an exon that is required for protein function [6]. Alternatively, they can be used to restore the translational reading frame to rescue the production of a therapeutic protein [7] as with dystrophin in Duchenne muscular dystrophy (DMD) [8,9,10] or to switch to an alternative beneficial isoform. Another possible application is using ASOs for transcriptional gene activation by either masking miRNA target sequences on an mRNA transcript or targeting target repressors such long non-coding RNAs [11,12]. Unfortunately, major effort for the treatment of central nervous system (CNS) disorders with ASOs are still needed. The fact that modified ASOs do not readily cross the blood–brain barrier (BBB), require highly invasive routes of administration, such as intrathecal or intracerebroventricular, placing a great burden on patients. However, promising strategies of systemic brain delivery across the BBB based on glucose-coated polymeric or lipid nanocarrier have recently emerged.

It is clear that ASOs are extremely flexible molecules that can be used to modulate most cellular processes. Their application in basic and translational research represents a potent tool which scientists can use to disentangle complex molecular mechanisms and to pave the way for new, and potentially revolutionary, therapeutic approaches for diseases that are currently considered incurable.

## 2. Evolution of Chemistry and Properties of ASOs

Antisense oligonucleotides (ASO) are short synthetic DNA or RNA molecules typically designed to target both coding and non-coding RNA in order to correct their expression level and ultimately their activity. They act by sequence paring so they need to be complementary to the target RNA to ensure sequence specific hybridization. The length is usually between 12 to 25 nucleotides, with a vast proportion of them designed to be around 18–21 nucleotides. Statistical calculations demonstrate that given the size of the human transcriptome, a sequence of 13 nucleotides for RNA occurs only once. Considering this fact, the average length of ASOs is enough to target a unique region of the genome [13].

**Figure 1:**
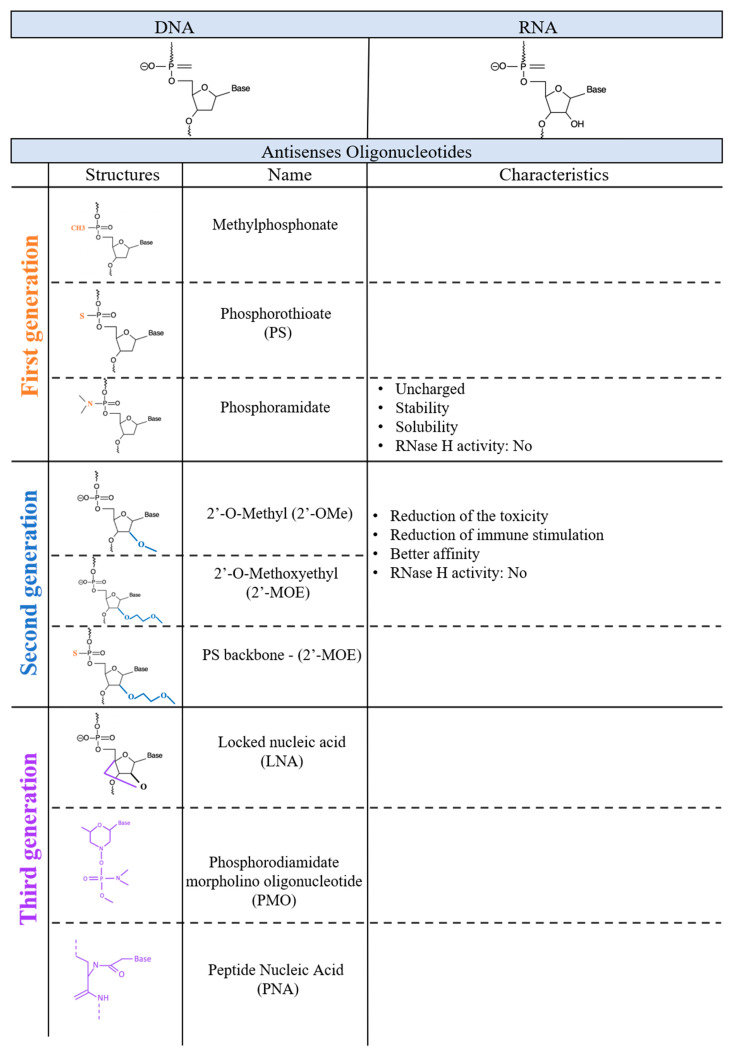
Schematic representation of different chemical characteristics of each generation of ASOs. Main chemical structure and specific characteristics for each compound described in the text are presented.

The first evidence of an injection of DNA inhibiting RNA activity was shown back in 1977, by impairing the translation of mRNAs in a cell-free system [14]. A year later, the first antisense oligonucleotides used in vivo were created [15]. Zamecnik and Stephenson generated a synthetic 13- nucleotide DNA molecule that targeted a Rous sarcoma virus ribosomal (35S) RNA. Targeting this RNA inhibited its translation and prevented the oncogenic transformation of chicken fibroblasts infected by this virus [15]. Following this seminal and very innovative first study, the use of antisense oligonucleotides increased dramatically. This was in large part due to the improvements of the chemical modifications which led to the production of novel classes of ASOs based on different and unique chemical structures [16]. Examples of different chemical modifications will be discussed in the following section and are presented in Figure 1.

## 3. First-Generation ASOs

Initially, ASOs were developed and used primarily to achieve translational silencing. They induce silencing through mediation by mRNA decay which leads to a reduced expression of the protein coded from the specific mRNA. This process is mediated by RNase H activation. RNase H is a ubiquitous enzyme that is able to recognize and cleave RNA-DNA duplexes that are at least 5 nucleotides long. Some ASO-RNA duplexes can recruit and activate RNase H, which leads to the degradation of the target RNA with an efficacy of up to 80–90%. In order to design a potent ASO for such applications, it is necessary to consider that different chemical modifications have different intrinsic abilities in recruiting RNase H [17,18,19].

The first ASOs used were synthetic deoxyribonucleotides, but their efficacy and application were limited due to several parameters that were overcome with chemical modifications. First generation ASOs, based on naked RNA, were highly sensitive to the degradation by both endo and exo-nucleases. These molecules have a limited ability to permeate into the membrane, and their solubility is low. Furthermore, the product of their degradation (phosphodiester oligonucleotides to dNMP2 mononucleotides) can be cytotoxic [17]. All of these characteristics taken together made first generation ASOs a questionable tool for research purposes and drug development. Therefore, in order to make them usable as a therapeutic or research tool, they needed to be chemically modified. The challenge was to improve their stability and efficacy while reducing their toxicity without reducing specificity. In the time since the first proof of concept studies involving the development of the first-generation ASOs, researchers have significantly improved the biochemical aspect of this technology.

## 4. Second-Generation ASOs

The first important advancement that led to the evolution of first-generation ASOs into second-generation ASOs was the substitution of one of the non-bridging oxygen atoms by different chemical groups which improved the stability of the molecule. Substituting the non-bridging oxygen with methyl, sulfur and amine groups led to the production of methyl-phosphonates, phosphorothioates (PS), and phosphoramidate respectively. These new backbones increased the overall oligo stability even though there were some substantial differences between them. The methylphosphonate backbone is a non-charged molecule for which the modification occurs on each nucleotide of the chain. The uncharged state of those molecules reduces the solubility and cellular uptake in that it permeates cells mainly by endocytosis and not by diffusion through the membrane. This chemical modification plays an important structural role because the absence of charges eliminates the charge-charge repulsions. This is what is normally observed in an RNA-DNA duplex leading to a less stable bond between the ASO and its target. Moreover, oligonucleotides with methylphosphonate backbones are not able to engage with RNAse H activity [20,21,22,23] and they cannot promote degradation of the target RNA. Therefore, despite the high stability of these molecules, they do not seem to be the ideal candidate.

PS-based oligonucleotides seem to have most of the characteristics that researchers are looking for in an ASO, and they have in fact been used as antisense therapies in multiple studies. The first characteristic is their ability to recruit RNase H and trigger its activity. These negatively charged ASOs are not fully resistant to nucleases but have a higher stability compared to phosphodiester linkages and they are highly soluble. Thus, PS ASOs are very useful for basic research studies, but more importantly, they are very suitable as therapeutic molecules.

The third member of first-generation ASOs are the phosphoramidates. Members of this family are uncharged molecules that do not activate the RNase H activity [24,25]. They are characterized by the presence of an NH2 group in position 3, which gives this chemical group the effect of increased solubility and enhanced resistance to nucleases [24,26]. Few studies have been conducted using this class of molecules (Agrawal and Gait, 2019). In one study, Agrawal and colleagues compared the efficacy of a phosphoramidate ASO targeted to HIV-1, thus inhibiting viral-induced syncytium formation and the expression of a viral protein. They did not report a significant difference of efficacy between phosphoramidate and PS [27]. Later, GRN163L, a 13 mer ASO containing all-phosphoramidate internucleotide linkages was created and tested in a clinical trial. It was found to be a potential cancer treatment which inhibited telomerase activity [28].

Despite the efficiency of some first-generation ASOs, PS backbone oligonucleotides are able to interact with both plasma and cellular proteins which lead to off-target effects. It is also important to highlight the fact that they are not fully resistant to RNases, a point that might be a strength in certain situations but may be a weakness in others. Furthermore, their chemistry is difficult to manipulate and functionalize, preventing the whole class of compounds to have the flexibility that scientists would like to see in a research tool. In order to overcome the weaknesses of the first-generation ASOs and develop ones that are excellent therapeutic molecules, scientists developed more advanced molecules that fall under the umbrella of second-generation ASOs.

Members of the second generation are characterized by the replacement of the hydrogen atom at the 2′-position of the ribose by an O-alkyl group leading to the formation of 2′-O-methyl (2′-OMe) and 2′-O-methoxyethyl (2′-MOE) nucleotides [29]. These molecules have several advantages over first-generation ASOs including the fact that they are less toxic, do not cause any robust immune reaction, are more resistant to nucleases’ degradation, and show a higher target engagement. Their mechanism of action is based on translation control of the target mRNA rather than RNase H-mediated target degradation. The peculiar mechanism of action characteristic of those molecules has not been described in full detail yet, but their ability to inhibit messenger translation was previously demonstrated in a polysome profiling experiment. It was shown that cells treated with ASOs are able to target a specific mRNA and show a strong accumulation of the target mRNA in the 40S and 60S subpolysome fractions, while the same messenger was associated with monosome and polysome fractions in cells treated with scramble oligos.

In order to increase the resistance to nucleases, a phosphate group can replace an oxygen atom as in PS ASO: an 2′-O-methoxyethyl (2′-MOE) with a PS backbone. To improve the mRNA decay, it is possible to induce the RNase H activity using different strategies, which led to the third generation of ASOs.

## 5. Third-Generation ASOs

The third generation of ASOs have a high degree of heterogeneity and involve many types of chemical modifications. Here we will present the most common of these modifications. The main goal of developing the next generation of ASOs was to improve resistance against nucleases and to increase their specificity. Thus, they are almost fully resistant to degradation by nucleases and peptidases, and this gives them tremendous stability in biological fluids [30]. In addition, their affinity for the target is much higher compared to members of the second generation.

Common among third-generation ASOs are Lock Nucleic Acids (LNAs) which are characterized by the presence of a methylene bridge connecting the 2′-oxygen and 4′-carbon of the ribose ring. This extra bond locks the flexibility of the parent furanose and increases the strength of their target interactions. The presence of a single LNA monomer is enough to increase the melting temperature of the RNA/ASO binding from +3 to +11 °C compared to a full DNA oligo. They can bind on a single- or double-strand DNA or RNA and form a stable duplex or triplex [4]. Compared to MOE, LNAs can increase the mRNA decay of their targets by up to 5 fold in mouse liver [31]. Despite their great power, some of them can induce a significant hepatotoxicity [32,33] which seems to be connected with the specific oligonucleotide sequence rather than with the chemistry as a whole. Researchers have found sequences that are more prone to lead to off-target effects and have more hepatotoxicity than sequences that are more target-specific [30]. It was also demonstrated that such kinds of hepatoxicity can be prevented or reduced using bioinformatic tools used to predict possible off-target effects. LNAs are such potent molecules that short sequences (12 to 15 nucleotides) are enough for an LNA-based ASO to be more effective than a longer ASO composed using a lower affinity modification [34].

Second-generation LNAs can interact with PS DNA to enhance their properties or trigger specific mechanisms of action. LNAs were shown early on to be formidable compounds and have paved the way for all antisense therapies used today. The first ASO to receive approval by the Food and Drug Administration (FDA) in 1998 and by the European Medicines Agency (EMA) in 1999 was a member of this class, and was very effective in the treatment of cytomegalovirus (CMV) retinitis (the details of which will be discussed later in this manuscript). In immunodeficient individuals, CMV infection can be quite severe when compared to healthy individuals where the infection can often go unnoticed. Therefore, the indication of this treatment is specific to patients with acquired immunodeficiency syndrome (AIDS) to prevent a progressive destruction of retinal cells [35,36,37]. Vitravene was the standard of care for years until it was withdrawn in 2002 in Europe and 2006 in the USA (EMA: EMEA/12382/02; FDA document number: 2011-14164) because the tri-therapy used to treat AIDS has substantially decreased the prevalence of CMV infection in these patients.

Another member of the third-generation ASOs is phosphorodiamidate morpholino oligonucleotides (PMO) which consists of a nucleic acid where the five-membered ribose heterocycle is replaced by a six-membered morpholine ring. As PMOs are non-charged, they are not able to recruit RNase H [38]. Peptide Nucleic Acid (PNA) molecules consist of a nucleic acid mimic in which a pseudo peptide polymer backbone is substituted for the PMO backbone of DNA/RNA. PNAs have the addition of a chain of N-(2-aminoethyl) glycine units to which the nucleobases are attached via methyl carbonyl linker [39]. Both PMOs and PNAs are uncharged, but can be covalently conjugated to a charged molecule to promote cell permeability. One of the disadvantages of these molecules is that they are rapidly cleared via urine excretion which means, in terms of therapeutic potential, they need to be administered often which increases the risk of side effects. Tricyclo-DNA (tcDNA) is a DNA analogue which increases the duplex formed by the ASO and the target RNA. The most remarkable characteristic of tcDNA which distinguishes them from all other classes of ASOs is their capacity to diffuse across the blood–brain barrier [40]. TcDNA is uncharged and, like PMOs and PNAs, they are unable to recruit RNase H. However, tcDNA has been recently used in the form of a gapmer to silence the HTT transcript [41].

Both LNAs and PMOs are uncharged molecules which decreases their affinity to plasma protein and increases the distribution and elimination in urine [42]. Only 10–30% of the amount administered is eliminated which leads to a high distribution in organs and thus a high bio-availability [43].

The chemical modifications made over the last decade have allowed ASOs to be highly specific, more stable, reliable, and safe. They can be selective for a single-nucleotide polymorphism (SNPs) using different modifications such as 2-thiothymidine, 3′-fluorohexitol nucleic acid, and cET in combination with a little gap to engage RNase H [44,45]. The affinity of the target is a crucial parameter for the design of an ASO. An improvement in affinity will improve the specificity, which results in reduced off-target effects which is one of the primary drawbacks of ASOs. The binding affinity of an ASO must be strong enough to disturb the secondary structure of the target RNA or compete with a potential RNA binding protein (RBP). ASOs require a delicate equilibrium in which they need to be very specific. However, overemphasis on the affinity for the target can lead to a low release of the cleavage products which can then result in a low turnover between target binding-cleavage and release of the product. Ultimately, this reduces their overall efficiency [46]. In order to improve the specificity of ASOs, it is possible to use bioconjugation to achieve better targeting. The delivery (which will be discussed in detail in the following section) can be enhanced via the conjugation of multiple moieties which can promote the tissue or cell specificity and reduce the clearance. For instance, ASOs can be linked to lipids such as cholesterol in order to facilitate the interactions with lipoprotein particles. ASOs can also be linked to peptides, sugars, or antibodies to address to a specific cell type [5].

As previously described, the activity of RNase H is not possible for every ASOs, and we are able to divide them based on that fact. However, while this inability may appear as an inconvenience, scientists have taken advantage of this characteristic by developing steric blockers to correct splicing or to compete with a miRNA binding on a specific target [16].

## 6. ASO Design

The strength and stability of interactions between an ASO and the complementary target RNA depends on many factors. Therefore, several considerations must be taken into account in order to have a successful ASO design. These include prediction of RNA secondary structures [47,48], identification of preferable RNA secondary local structures [49], motif determination and GC content calculations, and binding energy predictions.

Typically, the optimal length of an ASO to target a unique sequence is approximately 18–21 nucleotides. Shorter sequences increase the probability of off-target hybridization while longer sequences reduce the cache of cellular uptake [50], an aspect that is particularly crucial for all applications that are based on gymnotic uptake of unconjugated LNAs. Though an ASO should hybridize to any region of a target RNA sequence, secondary and even tertiary structures could likely result in some inaccessible site-to-ASO hybridization. Using free computational algorithms, like *m*fold [51] and *s*fold [52], allows for accurate predictions of all possible optimal target transcript secondary structures based on G, leading to effective ASO design. Once a non-folded region has been identified, the hit rate of potent ASO design can be further increased by targeting highly-conserved accessible motif [53], mapped by means of RNase H/chimeric oligodeoxynucleotide (ODN) libraries [54] or software based on mRNA accessible site tagging (MAST) [55]. The sequence is pivotal when it comes to ASO design and over the past several years, we have discovered a few crucial details that can increase the antisense activity just by designing the sequence correctly. Even if the mechanism and details of these characteristics are still under investigation, ASOs containing the CCAT, TCCC, ACTC and GCCA motifs have been shown to correlate with enhanced antisense efficiency [56]. As RNase H activity appears to be sequence independent, it seems to be strongly correlated with GC content, which then improves thermodynamic stability [57]. Moreover, ASOs should be checked not only for the presence of such motifs, but also for being self-complementary, which may cause dimer formation and therefore interferes with antisense hybridization.

In addition, to obtain a highly potent lead compound, the binding energy between ASOs and mRNAs needs to be considered, and indeed several programs are able to calculate their thermodynamic properties [47,58]. A BLAST search can also be performed on the final ASO sequence to ensure any off-target hybridization is not confounding. Hence, high RNA-binding affinity is a driver for higher ASO potency, however, many other factors are crucial, including ASO chemical design, RNase H recruitment, tissue/cellular uptake, and tissue distribution. To be therapeutically effective, systematically-injected ASO must resist nuclease degradation, exceed liver and renal clearance, escape non-productive sequestration by certain plasma protein, cross the capillary endothelium of a specific tissue, and penetrate the target cells in order to reach the active site at a desired concentration to produce a significant biological effect.

An unmodified ASO is susceptible to rapid break down by exo- and endonucleases in the body, and their overall charged properties confer a weak permeation across biological membranes. Therefore, ASO delivery represents the major translational challenge, unlike their use in preclinical research. In vitro, it is relatively easy to overcome all of these problems by enhancing ASOs’ internalization through transient permeabilization of plasma membrane by mechanical approach (electroporation, shockwave and ultrasound wave), or by means of a delivery cationic liposome system (Lipofectamin or Transfectam). Tiny ASOs, typically 15 nucleotides or less, exhibit the property of being internalized by gymnotic uptake. This aspect is double-sided because it does not require any further chemical modification, but at the same time, specificity to a given tissue or cell type is very difficult. These are some of the main reasons why, nowadays, most antisense therapeutics have focused on their topical use and delivery via the liver. However, chemical modifications represent some of the most effective approaches to improve their pharmacokinetic and pharmacodynamic properties. They include backbone modification, different ribose, base modification, and functionalization of the ASOs with a specific targeting motif, like Gal-Nac [59] or aptamers [60,61,62], against specific surface markers.

As previously discussed, PS-ASOs are easy to synthetize and carry a negative charge which allows for cellular delivery. Therefore, they might be designed in a way that makes them capable of activating RNase H and providing optimum protein-binding, resulting in suitable pharmacokinetics. The interaction of PS-ASO with 95% of plasma proteins extend their tissue elimination half-life, thus supporting peripheral tissue distribution. However, they also trigger off-target effects such as immune stimulation and complementary activation. Despite these disadvantages, PS chemical modifications are widely used for loss-of-function studies in vitro and in vivo for gene target identification and validation. However, PS modifications decrease the melting temperature (T_m_) which makes them inadequate for most therapeutic purposes. This can be overcome by combining additional chemical modifications, like the 2’ modification (2’OMe, 2’-MOE) and furanose ring modification (PNA, PMO, LNA), which allows for their comprehensive use in almost all ASO drugs or drug candidates in clinical settings. Moreover, while these modifications dramatically improve RNA-binding affinity, it is important to note that PS do not support RNase H cleavage, thus damping the efficacy of the ASO. Consequently, second- and third-generation sugar modifications are often implemented in a ‘gapmer’ design in which a central gap region, consisting of approximately 5 to 10 PS-modified 2’deoxynucleotides, allow RNase H to sit and promote RNA degradation. This gap is flanked on both 3’ and 5’ sides by approximately five modified nucleotides’ ‘wings’ (2’OMe or 2’-MOE) resistant to nuclease cleavage. Furthermore, the symmetry of the gapmer is not an indispensable condition. Indeed, nucleotides responsible for the RNase H engagement can be placed anywhere in the oligo sequence and they will still be able to engage RNase H enzymatic activity [30].

This strategy is effective with LNAs which exhibit high RNA-binding affinity due to incomparable T_m_ values. LNAs can be designed as ‘gapmer’ or ‘mixmer’. In a gapmer, two LNA segments are flanking a central ‘gap’ DNA segment whereas in a mixmer, LNA and DNA nucleosides are interspersed throughout the sequence of the oligonucleotide. While the gapmer design provides highly efficient RNA cleavage, the mixmer design cannot support RNA cleavage. However, because the LNA/RNA bond is very strong, it acts as an extremely efficient steric hindrance to change expression profile without destroying the RNA target [4]. Therefore, to inhibit mRNA expression and protein translation, designing the LNA as a gapmer is the most appropriate method. The scientific community is still debating about whether the recruitment of RNase H enzyme by the central DN/PS segment occurs only in the cytoplasm or if it also occurs in the nucleus. Recent data seems to strongly support the idea that this enzyme activity is detectable in both cellular compartments [63], allowing gapmer-PS-ASOs to target nascent RNAs while they are still in the nucleus. This property is crucial because it allows researchers to design ASOs against both exons and introns, resulting in the unique ability to modulate all steps of mRNAs’ processing, including ncRNAs [64,65,66]. Mixmer can also be used to inhibit translation but to do so it must be designed to bind to either the translation start site, thereby blocking binding of ribosomal subunits, or the region near the 5’ end of the pre-mRNA preventing 5’cap formation. In the case of pre-mRNA splice-switching modulation or ncRNAs’ inhibition, mixmer represents the preferred option by potentially creating both loss of function and gain of function phenotypes. Inhibition [67] or miRNA sequestration by LNA mixmer hybridization is a fairly recent discovery used to increase translation, conferring a wide therapeutic potential. MiRNAs’ inhibition via ASO targeting might represent a promising therapeutic approach because it allows for the de-repression of multiple mRNA simultaneously using a single compound. Today, the modulation of different genes or pathways requires a specific compound for each individual target.

Even if the knockdown at the RNA level is precluded, PMOs are also widely used with several drug candidates as splice modulators or steric block translational inhibitors. As PMOs as well as PNAs are uncharged molecules, this means that they have lower plasma protein binding which reduces the likelihood of non-specific interactions. A major disadvantage is that they are quickly cleared via urinary excretion, yet their charge-neutral chemistry gives them the possibility to be conjugated to cell-penetrating peptides (CCPs) and create PPMO [68], allowing for tissue-specific/cell-delivery treatment for various diseases.

Any base with PS or PMO substitution creates a chiral center, meaning that two possible stereoisomeric forms with different properties like hydrophobicity/ionic character, nuclease resistance, target affinity, and RNase activity [69] might exist. Today, nearly all ASOs are synthetized without stereo definition of the PS linkage, so the drugs exist as random mixtures of two stereoisomers. However, the identification of the most active stereoisomer enables breakthroughs in ASO drug development, allowing for more efficacious stereo-defined compounds at lower doses.

ASOs can be used with different routes of administration: intravenous, subcutaneous, intravitreal, intrathecal or local. It depends primarily on the targeted cell types and the chemical attributes of the molecule [70]. The pharmacological profile of ASOs are determined by its protein-binding capacity. One advantage of PS-ASO is their high plasma protein affinity, which increases systemic distribution allowing for a large uptake in tissues and cells. However, this also contributes to the acute toxicity, so the main challenge is to retain a good distribution while also decreasing toxicity [42,71]. Uncharged ASOs, like PNAs and PMOs, are rapidly excreted in the urine leading to a decrease in absorption. To increase their absorption, it is possible to couple them with peptides and create PPMO [68]. To obtain a systemic effect, the intravenous administration is the best route. Highly irrigated organs (liver, kidney, spleen) are easier to target than cardiac muscle, lung, and skin which are weakly irrigated. Currently, new therapeutic approaches are under investigation to increase bioavailability and biodistribution. For example, coupling ASOs to a fatty acid chain [72] or a peptide (Pip-6 PMO) is a winning strategy to increase bioavailability in muscle [73]. An alternative to the intravenous route can be subcutaneous delivery, which allows patients to self-administer after instruction by healthcare staff [74].

Given the localization of cells to be treated, local administration can be an effective means of treatment. Formirsen (described further), which is used to treat the retinal cells infected by Cytomegalovirus (CMV), was injected intraocularly into the vitreous humor. This route of administration offers the advantage of high cellular specificity, and reduces the elimination that occurs with systemic administration. However, in the case of Formirsen, there were severe localized adverse effects such as increased intraocular pressure and local inflammation [75]. For the central nervous system, intrathecal administration can be used to cross the blood–brain barrier. This route of administration ensures a significant bioavailability in the spinal cord and brain without renal elimination, hepatic elimination, and systemic exposure which can be a source of toxicity. However, given the invasiveness of this route, it is restricted to neurological diseases [76,77]. Nebulization (inhalation) is a method of administration that can be used to target pulmonary disease, and has the advantage of being non-invasive and allows for self-administration [78,79].

Other than implementing chemical modifications to improve drug-likeness, covalent conjugation to cell-targeting or cell-penetrating moieties and nanoparticles’ formulation were developed to address the delivery challenge. Implementing bioconjugates including lipids, peptides, antibodies, aptamers, and sugars allow for receptor-associated cellular- binding for specific tissue/cell uptakes. They are typically chemically well-defined and small, meaning that high-scale synthesis at a low cost is obtainable along with a favorable distribution profile. For instance, Gal-Nac conjugation is one of the leading strategies to enhance PS-ASO hepatocyte delivery given its high affinity to ASGR1 and ASPGR [5,71,80,81,82,83,84,85]. Antibodies [86] and aptamers are smaller in size with lower immunogenicity and are inexpensive to synthesize. They are widely used to enhance specific cellular- or tissue-targeting [87,88]. Peptides are an attractive source for ligands and there has been a recent interest in exploring their potential [89,90,91]. As mentioned before, chemical conjugation of CCPs with PMO generate PPMO which are highly useful for disease treatment, most notably for dystrophin splice-switching in the context of DMD [92,93]. It was recently demonstrated that conjugation of an ASO gapmer with glucagon-like peptide 1 (GLP-1) dramatically increased specific uptake from pancreatic B-cells, a particularly challenging organ in terms of delivery [94]. A wide range of nanocarriers that are able to cross biological membranes and provide nucleic acid protection from nuclease degradation are at various stages of development. The most common approach is lipid-based formulation (liposomes and lipoplexes). Their large size prevents renal filtration and allows for the delivery of a larger payload. A gene-activating ASO, MTL-CEBPA, delivered via lipid nanoparticles’ formulation [95] is currently under development for the treatment of hepatocellular carcinoma and cirrhosis [96,97]. Lipid carriers functionalized with peptides, PEG, or other ligands for cell-specific targeting are also possible. However, increasing the complexity could also increase the toxicity which limits their clinical use. Therefore, biological nanocarriers such as exosomes have attracted growing interest, especially due to the safe nature of administration. Efficient loading of therapeutic nucleotides remains extremely elusive and, as of now, is the limiting factor for this delivery method. During the discovery phase, combinations of molecular modifications with conjugation or complexation strategies makes it possible to extend the range of suitable pharmaceutical targets. It also allows for specific ASO drug design with larger therapeutic index to treat patients with rare or currently untreatable diseases.

Animal models have been essential for proof of principle studies as well as for pharmacokinetic and pharmacodynamic investigations. Treating mice model with ASO was proven very effective to collect information and data on drug metabolism and target engagement. If an ASO is designed to target a specific tissue, typically with one of the strategies just described, an animal model is important to provide information about safety and efficacy. However, when an ASO is designed for a non-coding target in humans, it might be ineffective in a mouse model since most of the non-coding RNA are not very well conserved between different species. In the specific case of miRNA inhibition, even when the target miRNA is conserved between species, the results of treatment with ASOs might be profoundly different because the miRNA networking can be completely different moving from one species to another. These targets are specific to the transcriptome of each species and will result in different effects (including side effects), therefore, safety might become a major concern.

## 7. ASO-Based Therapeutics

Thanks to the improved chemical modifications in these molecules which make them more stable and more specific, ASOs have become a very powerful therapeutic tool. Since the first ASO was developed for a therapeutic purpose (created by Zamecnik and Stephenson in 1978 [15]), ASOs have been widely used, so much so that some of them have received an FDA approval and are now present on the market [98], the most relavant ones are described in the next section and listed in Table 1.

## 8. FDA Approved ASO-Based Pharmaceuticals

### 8.1. Formirsen (Vitraven^®^)

In 1999, Formirsen (Vitravene^®^) marketed by Ionis Pharmaceuticals (formerly Isis) and Novartis Ophthalmics received the first ASO FDA approval. A year later, the EMA gave the same authorization. It is the only first-generation ASO to have received approval. It is a PS oligonucleotide targeting the viral mRNA encoding the early -2 (IE-2) protein, required for viral replication. Its indication was for the treatment of the cytomegalovirus (CMV) retinitis. CMV infection is of great concern in immunosuppressed individuals, while in healthy individuals, it often goes unnoticed. So the treatment was recommended for acquired immunodeficiency syndrome (AIDS) patients because a CVM infection could lead to the progressive destruction of the retinal cells [35,36,37]. The prevalence of the CMV infection in AIDS patients was reduced considerably thanks to the tri-therapy; so based on this, and for commercial reasons, Vitravene was withdrawn from the market in Europe and the USA (EMA: EMEA/12382/02; FDA document number: 2011-14164). It is worth noting that this withdrawal was not related to any safety concerns.

### 8.2. Mipomersen (Kinamro^®^)

Perhaps one of the most well-known ASOs, approved by the FDA in 2013, is Mipomersen (Kinamro^®^) developed by Grenzyme. This ASO is composed of PS-modified nucleotides surrounded by 2′O-MOE-based nucleotides, so it is a gapmer that belongs to the second generation. It is used to treat homozygous familial hypercholesterolemia patients. This pathology is caused by the mutation of the LDL cholesterol receptor, the pro-protein convertase subtilisin/kexin 9 (PCSK9) or apolipoprotein B (apo B), leading to the elevation of the low-density lipoprotein (LDL). This deregulation of LDL level results in an increased risk of developing coronary heart disease and atherosclerosis in young people [99,100,101].

### 8.3. Patisiran (Onpattro^®^)

Patisiran (Onpattro^®^), is the first siRNA-based molecule that received the AMM. Developed by Alnylam, it targets the transthyretin (TTR) protein involved in the transport of the thyroxin and retinol-binding protein vitamin A complex. Patients who suffer from the hereditary transtherthyretin-mediated amyloidosis (hATTR) present with a mutation in a TTR gene leading to the production of an abhorrent TTR which is more susceptible to misfolding. This generates TTR amyloid fibril in the extracellular space of the heart, liver, nerve, and gastrointestinal tract which can disrupt the normal working of the organs. After the release of this siRNA in the cytoplasm of cell, it interacts with TTR mRNA leading to the reduction of TTR protein expression and the inhibition of the fibrils’ formation [102].

### 8.4. Inotersen (Tegsedi^®^)

Inotersen was developed by Ionis Pharmaceuticals in 2018. It also received FDA approval for hATTR treatment. Similar to Patisiran, Inotersen aims to decrease the TTR protein expression. It consists of a gapmer composed of a sequence of PS oligonucleotides surrounded by five 2′MOE nucleotides. Although Inotersen received FDA approval, special renal surveillance is required because there is a risk of thrombocytopenias and glomerulonephritis [74].

### 8.5. Givosiran (Givlaari^®^)

Givosiran was developed by Alnylam Pharmaceuticals, and is the second siRNA approved by the FDA for the treatment of acute hepatic porphyria (AHP) which is a genetic disorder characterized by the excessive production of delta-aminolevulinate syntethase 1 (ALAS1). This over-expression of ALAS1 leads to the accumulation of neurotoxins such as aminolevulinic acid (ALA), and porphobilinogen (PBG) which causes abdominal pain, nausea, and seizures [103]. To improve the specific delivery to the liver, this siRNA is conjugated to Gal-Nac in order to decrease ALAS1 [104].

## 9. New Drugs Currently under Investigation

Currently, there are a number of candidates under investigation that are seeking FDA approval. We focus here on only a few examples that are in Phase 3 clinical trials and that are showing promising results.

### 9.1. Vutrisiran

Vutrisiran is an ASO developed by Alnylam Pharmaceuticals with the same indication as Patisiran. It is under investigation in Phase 3 clinical trials for the treatment of hATTR. It is also an siRNA that targets TTR mRNA to decrease the production of the protein [105].

### 9.2. Tominersen

Tominersen is a new promising drug for the treatment of Huntington’s disease. This neurodegenerative disease is caused by the mutation of the coding gene of the huntington (HTT) protein. It corresponds to an increase in the number of repetitions of the CAG triplet leading to a mutant HTT with extended polyglutamine tract. This mutated protein causes the accumulation in neurons and affects their normal functioning which results in dystonia, cognitive dysfunction and behavioral difficulties [106,107]. Torminersen is developed by Ionis Pharmaceuticals and targets the mutated HTT mRNA in order to decrease protein expression. The ASO showed a good tolerability without adverse effects even with high doses [70]. The Phase 3 enrollment has recently been completed (clinical trial ID NCT03761849).

### 9.3. Volanesorsen (Waylivra^®^)

Volanesorsen was developed by Ionis Pharmaceuticals, and is indicated for familial chylomicronemia syndrome. This syndrome is caused by a mutation of the lipoprotein lipase (LPL) gene leading to a poor fat breakdown. It results in an accumulation of fat in the blood and causes abdominal pain, eruptive xanthoma, pancreatitis, hepatosplenomegaly, and lipemia retinalis. Volanesorsen targets APOC3 mRNA in order to decrease plasma triglyceride and apolipoprotein C-III levels [108]. Results from preliminary clinical trials were so promising that a conditional marketing authorization was approved in Europe (EMEA/H/C/004538/0000).

### 9.4. Miravirsen

In contrast to the previous examples, Miravirsen does not target an mRNA but rather a microRNA (miRNA). MiRNA are short non-coding molecules (22 nucleotides) that target RNA by sequence complementary. MiRNA-bonding induce mRNA decay and the inhibition of the translation, and because they are involved in numerous biological processes, their deregulation is implicated in many pathologies [109]. Miravirsen was developed by Roche/Santaris for the treatment of hepatitis C infection. Its target, miR-122, is crucial for the correct replication of the virus [110] and is liver-specific, thereby insuring the organ specificity of the drug. Miraversen is a third-generation ASO, composed of LNA and PS modification. It is fully complementary to mature miR-122, but also to the precursors of miR-122 (pre-miR-122 and pri-miR-122), leading to the reduction of miR-122 production. The Phase IIa trials have shown a reduction of viral RNA-level but not a complete disappearance of the miRNA [111].

Another anti-miR-122 (RG-101) was developed by Regulus Therapeutics. The chemical modification of nucleotides differs from Miravirsen. It is composed of phosphorothioate coupled to an N-acetylgalactosamine group (Gal-Nac) in order to increase the hapatocytes’ uptake due to the presence of asialoglycoprotein receptor at the surface of the cells. Unfortunately, the development of the molecule was halted due to liver toxicity [112].

## 10. ASO Application in a Pandemic World

Severe acute respiratory syndrome coronavirus 2 (SARS-CoV-2) is the cause of the ongoing coronavirus disease 2019 (COVID-19) pandemic, which is currently creating a world health emergency [113]. Although huge global efforts have been made to understand SARS-COV-2 and develop vaccines [114], no effective antiviral treatments have been readily available, with just Remdevisir authorized by the US FDA for emergency use [115]. To date, considered therapeutic options rely on symptomatic and supportive care. Several drug candidates have already been approved [70,116] and many are in clinical trials for other diseases including Marburg virus (AVI-7288) and Ebola virus (AVI-7537) [117]. ASO-based therapy has great potential in combatting outbreaks of SARS-CoV-2 due to their excellent target-specificity, dual mechanism of action, easy design, rapid development, low toxicity, and relatively low production cost [3]. It should also be noted that their use is well suited to targets that present a high mutation rate, like any newly emerging viruses or mutant variants of known viruses. Thus, ASOs may be best utilized by targeting specific variants, as they are well suited for target-specificity.

The potential for ASOs as virus inhibitors dates back to 1970, with the first antisense experiment utilizing ASOs to block Rous sarcoma virus replication and the first antisense molecule approved by the FDA, Fomivirsen [118]. Moreover, many studies have demonstrated ASO efficacy against strongly pathogenic virus in infections such as Ebola [119], Influenza [120], Hepatitis B [121], Hepatitis C [122], and Japanese Encephalitis [123]. Indeed, the publication of three ASO patents as SARS-CoV infection treatments attest to this potential. Ionis Pharmaceuticals developed an ASO to disrupt the pseudoknot in the frameshift site of SARS-CoV (WO2005023083) [124], whereas AVI BioPharma, Inc., published an ASO targeting the 3‘terminal end of the negative strand of the viral RNA inhibiting their replication (WO2005013905) [117]. Another patent published by Stein David described ASO against ORF1 AUG (217–245 bp) of the viral genome (US20030224353) [125].

**Figure 2:**
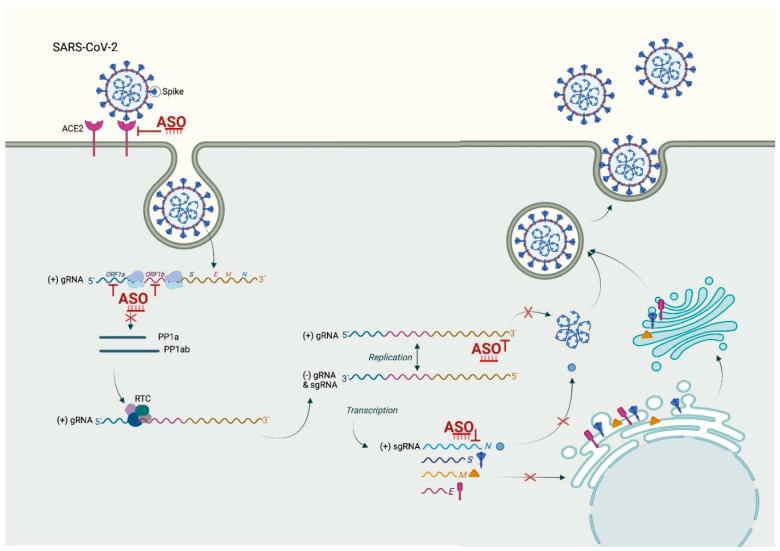
Possible therapeutic use of ASOs in the SARS-CoV-2 infection. ASOs can be used to prevent virus recognition though ACE-2 receptor and prevent SARS-CoV-2 from entering into the cell host. Moreover, ASOs can be used to stop the viral life cycle at different steps, providing important therapeutic entries. In addition, targeting different phases of the viral life can allow combinational therapy to produce a stronger inhibition. This figure was created with BioRender.com; accessed date 12 Janaury 2022.

To rationally design an ASO, the knowledge of the viral genome sequence and structure is sufficient, and approaches that can inhibit the SARS-CoV-2 life cycle at different stages are important for COVID-19 management (Figure 2).

SARS-CoV-2 has a 29,903 base of single-stranded, positive-sense RNA flanked by 5′ and 3′ untranslated regions (UTRs) and shares 89.1% homology with SARS-CoV (GenBank accessible number MN908947). After infection, the viral genome is translated either in non-structural proteins (NSPs) from two open reading frames (ORF1a and ORF1b) taking part in forming the replication/transcription complex (RTC), or structural proteins from subgenomic viral RNAs. Among these, the most important are the nucleocapsid (N) protein, the transmembrane (M) protein, the envelope (E) protein, and the spike (S) protein [126]. As all of these are associated with the physiopathology and virulence mechanism of SARS-CoV-2, they are suitable targets for ASO. ASO-based therapy targeting transcript encoding of a viral protein or genomic RNA itself, which serve as a template for viral replication, could be developed as a response to the COVID-19 pandemic [127].

However, the viral RNA genome has a high mutation rate enabling it to rapidly evolve and adapt. This allows it to evade the immunity in response to previous infections or vaccinations by slightly modifying its core proteins [128,129], causing new outbreaks with more severe disease [130,131,132]. To date more than 80 variants have been identified in the spike protein that confer a fitness advantage and are spreading worldwide with an increasing transmission rate, thereby elevating it to a pandemic [133,134].

Certain RNA structures, crucial for the viral cell cycle, are well conserved amongst the genome. This makes them valuable ASO targets (Figure 2) to fight multiple viral strains and to overcome drug resistance and prevent virus escape, thus halting its global spread [135]. Among the most studied is the stem-loop 2 motif (s2m) which is found evolutionary conserved within the 3‘ UTR of many different RNA viruses, including SARS-CoV and SARS-CoV-2 [126,136,137], as well as in different patients who tested SARS-CoV-2-positive [138,139]. This makes it an attractive target for therapeutic intervention. The s2m motif is stably and highly folded in a three-dimensional structure with a few exposed bases and is present in all positive-sense transcripts, both genomic and subgenomic. LNA-based gapmer ASOs designed against the s2m were found to be capable of successfully degrading the RNA in a sequence-specific manner with an inhibitory effect on SARS-CoV-2 replication in vitro [140]. This offers a suitable lead compound and strong starting point for further therapeutic development in order to treat COVID-19 and other diseases caused by viruses harboring the s2m motif. However, when considering s2m as a target for virus inhibition, it is worth considering that while it is a remarkably stable genome element, some mutation can arise over time [138,139] and using a cocktail of ASOs designed to target multiple genomic regions, it might be a potential therapeutic strategy to guard against emerging resistance.

Recent advances in high-throughput structure probing strategies (SHAPE-MaP and DMS-MaPseq) allows for the characterization of the full-structural RNA landscape pinpointing a well-defined subset of highly-conserved and weakly-structured regions ideal for ASO design [141,142]. Using structure-disrupting, antisense locked nucleic acids (LNAs), Huston et al. demonstrated that RNA motifs within conserved well-folded regions play functional roles in the SARS-CoV-2 life cycle, but also that these ASOs have antiviral potential [143]. Of particular interest, a recent study based on in vivo click icSHAPE technology illustrates how the identification of conserved RNA structures and host RBPs that bind to SARS-CoV-2 RNA viral genomes represent vulnerable drug targets to fight the still-ongoing COVID-19 pandemic. Strikingly, treatment with ASO targeted RNA-conserved structures and predicted RBP binding sites, dramatically reducing SARS-CoV-2 infection in different human cell lines, suggesting effective approaches to combat viral disease [144].

Other LNA gapmer ASO candidates were also predicted to have excellent properties to target more key domains such as the 5’untranslated region (5’UTR), and the open reading frames 1a and 1b (ORF1a and ORF1b) involved in the expression of the RTC and gene N, which encodes the genome-associated nucleoprotein, with GAP1 candidate on 5’UTR being the most potent [127]. Furthermore, the possibility of administering ASOs directly to the lungs by aerosol makes them promising for the respiratory syndrome treatment [145,146,147,148]. Recently, Zhu et al. identified an intranasally-delivered LNA-ASO targeting the 5‘ leader sequence of SARS-CoV-2. It was found to be particularly effective in disrupting the highly-conserved stem-loop structure and potently suppressed viral infection, including those caused by variants of concern [149].

Another strategy to minimize the risk of escape-mutant occurrence is the inhibition of cellular genes that are unlikely to mutate, which the viruses depended on. The most forward approach is to silence the angiotensin-converting enzyme 2 (ACE2) as SARS-CoV-2 entry receptor [150,151]. Using splice-switching oligonucleotides (SSOs), it should be possible to modulate the alternative splicing of the ACE2 gene to remove critical domains required for the entry of SARS-CoV-2 into host cells (Figure 2). With the help of bioinformatics, new exons in the 5‘UTR, 3‘UTR, and intronic regions of the ACE gene likely to modulate the alternative splicing has been predicted. The inclusion of such novel exons in the 5‘UTR or intronic regions by means of SSOs leads to the exclusion of major ACE2 amino acid residues responsible for the interaction with the receptor-binding domain (RBD) of spike SARS-CoV-2 protein (Yan R, 2020). This prevents their binding and entrance into the host cells. Similarly, inclusion of exons in 3’UTR can remove the C-terminal transmembrane domain of ACE2, generating a soluble ACE2 isoform that competes with the membrane-bound form in virus binding without allowing entrance into the host cells [152].

Degraded structural protein RNAs could offer a variable strategy as well. In a recent study, Xiaoxuan Su et al. raised the possibility of designing a chimeric oligonucleotide comprising a 2’-OMe-modified antisense oligonucleotide. The purpose would be to recognize the target viral RNA and a 5’-phosphorylated 2’-5’ poly (A)4 (4A2-5-ASO) for guided RNase L activation to sequence-specifically degrade the viral RNA in order to target envelope and spike proteins thus inhibiting SARS-CoV-2 infection [153]. Additionally, it is important to consider the target RNA folding problem in ASO design, which leaves important structured targets inaccessible. However, developing a structure-based ASO design method would seem to provide 3D modified ASOs (PMO and LNA) that can invade specific binding pockets within well-conserved structures. This may result in a strong candidate for inhibiting SARS-CoV-2 replication [154,155].

As previously mentioned, the strong tendency to undergo frequent genetic mutations leads to a continuous emergence of new variants with enhanced infectivity, even among vaccinated people. Therefore, SARS-CoV-2 surveillance is also essential in controlling widespread transmission given the challenge in predicting the severity of illness in infected individuals [156]. Although the RT-PCR technique is currently favored for the detection of SARS-CoV-2, significant efforts have been made to improve and find new practical detection systems that can be used to promptly diagnose virus infection. Among them, colorimetric assay based on gold nanoparticles (AuNPs) capped with ASOs specific for N-gene may provide a virus detection within 10 min after RNA isolation [157], further highlighting the powerful flexibility of ASOs.

## 11. Discussion

The advent of sequencing and high throughput analysis in the last two decades has given us a better molecular understanding of pathologies and genetic characterization. This has notably led to the development of targeted therapies and a paradigm shift in how we view personalized medicine. Currently, genetic diseases, cancer, and neurodegenerative diseases are within the domain of ASO-based therapies, however, ASOs can also be used to fight viral infections. They are especially important now that we are in the midst of a major health crisis. ASOs are a powerful tool, and when combined with vaccinations, they have the potential ability to effectively fight the COVID-19 pandemic by providing a possible preventive and curative treatment.

ASOs are mostly known for degrading an mRNA or for modifying the splicing process. The molecules currently authorized by the governmental authorities or which are undergoing clinical trials act by one of these mechanisms. However, the versatility and adaptability of these molecules have made them excellent candidates for more innovative and original therapeutic strategies [16]. It is possible to take the advantage of the complementary sequence to mask different regions in order to induce the release of RNA-binding proteins [158] or protect RNA from miRNA degradation [159]. These examples have not been detailed in this review because the focus was on the degradation activity induced by ASOs. Here, we have emphasized the specificity of ASOs through sequence complementarity. However, it is possible to adopt another strategy that indirectly targets an mRNA. The idea would be to target an actor of the RNA metabolism such as quality control of the mRNA. Nonsense-mediated decay (NMD) was found to eliminate an mRNA containing a premature stop codon which would have led to the formation of an abnormally short protein [160]. In some cases, such as in a Duchenne myopathy, mutations cause the premature stop codon formation and therefore, a lack of the protein. Inhibition of one component of the NMD (UpF3b) results in the stabilization of the dystrophin mRNA in an MDX mouse model. It also restores the production of the factor IX protein in a hemophilia mouse model [161]. These ASOs act by recruiting and activating RNase H to induced mRNA decay. Contrary to previous examples, they do not target the abnormal mRNA, but act on RNA’s processing.

Several studies have developed new therapeutic strategies which induce the degradation of mRNA mediated by ASO without the involvement of RNase H. No-go-decay (NGD) is involved in translational control as it acts when the ribosome progression is disrupted, and induces the mRNA decay by endonucleolytic [162]. ASOs were designed to alter the ribosome progression and induced the NGD, thus the mRNA decays in an RNase H independent way [163]. Finally, the mRNA decay activity of ASO mimics the physiological RNAi mechanism carried out by the miRNA. Many studies have shown that miRNA can be sequestered mostly by circular RNA. It is possible to use ASOs to redirect miRNA to their target and induce mRNA decay by the miRNA [164].

Therefore, ASOs are an extremely versatile group of molecules used specifically to correct genetic alterations and propose a personalized therapy for each individual patient [1].

## Figures and Tables

**Table 1:** Drugs based on ASO technology that have already received FDA approval or are at the latest stages of clinical trials.

Drug Name (Company)	Company	Chemistry	Design (Mix-Gap)	Disease	Date FDA Approval Stage of Trial	Administration
Formirsen (Vitraven^®^)	Ionis Pharmaceuticals & Novartis Ophthalmics	PS & 2′O-MOE	First generation	Cytomegalovirus (CMV) retinitis	1999 (FDA approval)—withdrawn (2006 in USA)	Intravitreal
Mipomersen (Kinamro^®^)	Grenzyme	PS	2nd generation—Gapmer	Homozygous familial hypercholesterolemia	2013	Intravenous
Patisiran (Onpattro^®^)	Alnylam	siRNA	Double-stranded small interfering RNA encapsulated in a lipid nanoparticle	Hereditary transtherthyretin- mediated amyloidosis	2018	intravenous
Inotersen (Tegsedi^®^)	Ionis Pharmaceuticals	PS & 2′O-MOE	2nd generation—Gapmer	Hereditary transtherthyretin-mediated amyloidosis	2018	intravenous
Givosiran (Givlaari^®^)	Alnylam Pharmaceuticals	siRNA	siRNA—conjugated to Gal-Nac	Acute hepatic porphyria	2019	intravenous
Vutrisiran	Alnylam Pharmaceutical	siRNA	siRNA—conjugated to Gal-Nac	Hereditary transtherthyretin-mediated amyloidosis	Phase 3	subcutaneous
Volanesorsen (Waylivra^®^)	Ionis Pharmaceuticals	2’-MOE	2nd generation	familial chylomicronemia syndrome	Phase 3/EMA approved 2019	sub cutaneous
Miravirsen	Roche/Santaris	LNA & PS	3rd-generation anti-miRNA	Hepatitis C virus infection	Phase 3	ND
RG-101	Regulus Therapeutics	PS coupled to an N-acetylgalactosamine group		Hepatitis C virus infection	Phase 3	ND
Pegaptanib (Macugen^®^)	OSI Pharmaceuticals	Aptamer	Aptamer	Neovascular age-related macular degeneration	2004	intravitreal
Eteplirsen (Exondys 51^®^)	Sarepta Therapeutics	Phosphorodiamidate morpholino oligomer	3rd generation	Duchenne muscular dystrophy	2016	intravenous
Nusinersen (Spinraza^®^)	Ionis Pharmaceuticals, Biogen	PS & 2’-MOE	3rd generation	Spinal muscular atrophy	2016	intrathecal
Defibrotide	Jazz Pharmaceuticals	Mixture of single-stranded and double-stranded phosphodiester oligonucleotides	Aptamer	Veno-occlusive disease in liver	2016	intravenous
Inotersen (Tegsedi^®^)	Akcea Therapeutics	2’-MOE	2nd generation	Polyneuropathy caused by hereditary transthyretin-mediated (hATTR) amyloidosis	2018	subcutaneous
Milasen	Boston Children’s Hospital	PS & 2’-MOE	2nd generation	Mila Makovec’s CLN7 gene associated with Batten disease	2018	intravenous
Patisiran (Onpattro^®^)	Alnylam	PS & 2’-MOE	Gapmer	Polyneuropathy caused by hATTR amyloidosis	2018	intravenous
Golodirsen (Vyondys 53^®^)	Sarepta Therapeutics	PMO	3rd generation	Duchenne muscular dystrophy	2019	intravenous
Givosiran (Givlaari^®^)	Alnylam	Gal-Nac-2’OMe		Acute hepatic porphyria (AHP)	2019	intravenous
Viltolarsen (Viltepso^®^)	NS Pharma	PMO	3rd generation	Duchenne muscular dystrophy	2020	intravenous
Casimersen (Amondys 45^®^)	Sarepta Therapeutics	PMO	3rd generation	Duchenne muscular dystrophy	2021	intravenous

## Data Availability

Not applicable.
